# Two-year real-world experience with somatrogon in children and adolescents with growth hormone deficiency

**DOI:** 10.3389/fendo.2026.1685851

**Published:** 2026-01-28

**Authors:** Gianluca Tamaro, Chiara Rodaro, Alice Fachin, Antonella Fabretto, Gianluca Tornese

**Affiliations:** 1 Institute for Maternal and Child Health IRCCS “Burlo Garofolo”, Trieste, Italy; 2 Department of Medicine, Surgery and Health Sciences, University of Trieste, Trieste, Italy

**Keywords:** growth hormone deficiency, LAGH, somatrogon, real world, real life

## Abstract

**Introduction:**

Growth hormone deficiency (GHD) in children and adolescents is a chronic condition requiring long-term therapy with recombinant human growth hormone (rhGH). Daily injections pose adherence challenges, prompting the development of long-acting GH (LAGH) formulations, such as once-weekly somatrogon. While phase III trials have demonstrated its efficacy, real-world data are limited.

**Methods:**

This retrospective study evaluated all pediatric patients with GHD who initiated somatrogon between March 2023 and January 2025 at a tertiary endocrine center in Italy and completed at least 6 months of treatment.

**Results:**

Forty patients (50% naïve; 50% switched from daily rhGH) were included. At 6 months, height SDS increased significantly in both naïve (Δ +0.19) and switch patients (Δ +0.17), with no significant difference between groups. However, by 18 and 24 months, naïve patients showed significantly greater height gains, with a median cumulative Δ of +0.81 at 18 months. IGF-1 SDS increased significantly only in the naïve group. Median gain in height SDS at 12 months in naïve patients (+0.37) was lower than reported in registration trials, likely reflecting the broader clinical heterogeneity of real-world populations. Treatment was well tolerated, with no discontinuations and few mild adverse events. Several families reported improved adherence and quality of life.

**Conclusions:**

In this first real-world cohort, somatrogon was safe and effective in supporting linear growth, although height gains were lower than in clinical trials. Weekly administration may offer practical benefits, especially for patients with complex needs or poor adherence to daily injections.

## Introduction

Growth hormone deficiency (GHD) in children and adolescents is a rare but impactful endocrine disorder that affects growth, metabolism, and overall development. The advent of recombinant human growth hormone (rhGH), somatropin, has significantly improved the management of GHD, enabling near-normal growth and favorable metabolic outcomes for affected individuals (1). However, daily rhGH injections are associated with challenges in adherence, treatment burden, and patient satisfaction (2).

Long-acting growth hormone (LAGH) formulations have emerged as an innovative response to these challenges, reducing injection frequency while maintaining efficacy and safety (3, 4). Somatrogon, a novel long-acting GH analog, has been developed for once-weekly administration and ensures sustained GH activity. Clinical trials have shown that somatrogon is non-inferior to daily rhGH in terms of height velocity, safety profile, and metabolic outcomes (5–7).

While randomized controlled trials have established the efficacy of somatrogon under ideal conditions, data on its effectiveness in real-world clinical settings are still lacking (4). Real-world studies are crucial for understanding treatment effectiveness in routine clinical practice, as they account for the variability in patient characteristics, treatment adherence, and long-term outcomes that are often not fully represented in controlled clinical trials (8).

This study evaluates the first two years of real-world use of somatrogon in pediatric and adolescent patients with GHD, aiming to bridge the gap between clinical trial evidence and routine care by providing endocrinologists with practical insights into its effectiveness, safety, and dose management.

## Materials and methods

We conducted a retrospective observational study at the Institute for Maternal and Child Health IRCCS “Burlo Garofolo” in Trieste, Italy – a tertiary care and national reference center for pediatric endocrinology.

All patients diagnosed with GHD according to Italian guidelines, who initiated somatrogon between March 2023 and January 2025, and who received at least 6 months of treatment were included. According to the Italian national guidelines, the diagnosis of GHD was based on one or more of the following auxological/clinical criteria:

- height ≤ –3 standard deviation score (SDS);
- height ≤ –2 SDS and annual growth velocity ≤ –1 SDS for age and sex, assessed over at least 6 months, or a height decrease of ≥0.5 SDS/year in children older than 2 years;
- height ≤ –1.5 SDS relative to mid-parental height, combined with annual growth velocity ≤ –2 SDS or ≤ –1.5 SDS over two consecutive years;
- annual growth velocity ≤ –2 SDS or ≤ –1.5 SDS over two consecutive years, even in the absence of short stature, after exclusion of other pathological causes of growth failure;
- presence of hypothalamic–pituitary malformations or lesions on neuroimaging;

together with a peak GH response <8 ng/mL on two different stimulation tests using distinct stimuli (arginine and insulin, in our center) (9).

Data were retrieved from the “G2 Clinico” electronic health record system. Collected variables included sex, age at somatrogon initiation, pubertal stage, treatment status (naïve or previously on somatropin), and, for pre-treated patients, the reason for change, duration of prior therapy, and last somatropin dose (mcg/kg/day). In addition, GHD etiology was categorized as follows: 1) combined pituitary hormone deficiency (CPHD), defined by at least another pituitary deficiency other than GHD; 2) definite GHD (dGHD), defined by peak GH level of <8 ng/dL and the presence of an identifiable genetic, functional, or anatomical cause (such as a genetic diagnosis of isolated GHD, acquired damage, or the presence of hypothalamic or pituitary abnormalities on MRI); and 3) idiopathic GHD/short stature unresponsive to stimulation tests (SUS), defined as a peak GH level of <8 ng/dL without any identifiable genetic, functional, or anatomical cause (9). Etiological classification was based on clinical, biochemical, and radiological findings recorded at diagnosis.

Additional data included somatrogon starting dose (mg/week), weight (kg), height and BMI (expressed in SDS) (10), bone age (Greulich & Pyle method) (11), difference between bone age and chronological age, and IGF-1 SDS relative to chronological and bone age (12).

IGF-1 was measured using a chemiluminescent immunoassay (IMMULITE 2000, Siemens), with a sensitivity of 20 ng/mL. Measurements during treatment were taken 4 days after injection (13).

We collected data at 6 months of treatment for all patients, and at 12, 18, and 24 months for those with available follow-up. Collected variables included somatrogon dose and adjustments, height SDS, BMI SDS, IGF-1 levels, bone age, and adverse events. For the Switch group, data from the 6 months preceding the initiation of somatrogon were also collected. Changes from baseline (Δ) were calculated for height, BMI, and IGF-1 SDS.

This study did not require ethics committee approval per Italian data protection regulations (Authorization no. 9/2014). Parents provided written informed consent for clinical data use in research and education (14).

Data are reported as median and interquartile range (IQR) for continuous variables and frequency (%) for categorical variables. Between-group comparisons were made using the Mann–Whitney U test or Fisher’s exact test, as appropriate. Wilcoxon signed-rank test was used for paired comparisons over time. To explore predictors of growth response, we performed a multiple linear regression analysis with Δ height SDS at 6 and 12 months as the dependent variables. Analyses were conducted with jamovi (version 2.3.28), with *p* < 0.05 considered statistically significant.

## Results

A total of 40 GHD patients (40% female, 45% prepubertal) who received somatrogon treatment for at least 6 months were included in the study, with half classified as treatment-naïve (*Naïve* group) and half as having transitioned from prior somatropin therapy (*Switch* group). Regarding GHD etiology, 4 patients (10%) had CPHD, 11 (28%) had dGHD, and 25 (62%) had SUS. The main demographic and clinical characteristics of the overall cohort, as well as those of the Naïve and Switch groups, are summarized in **Table 1**.

**Table 1:** Demographic and clinical characteristics of patients treated with somatrogon, divided into two groups: Naïve (patients who had not received prior growth hormone treatment) and Switch (patients who transitioned from somatropin treatment to somatrogon).

Baseline	Total	Naïve	Switch	P-value
Number of patients, N (%)	40 (100%)	20 (50%)	20 (50%)	**-**
Female sex, N (%)	16 (40%)	10 (50%)	6 (30%)	0.197
Chronological age (years)	11.4 (10.0;12.9)	11.6 (10.8;13.1)	11.4 (7.2;12.8)	0.640
Prepubertal, N (%)	18 (45%)	7 (35%)	11 (55%)	0.204
Bone age (years)	10.5 (7.9;12.0)	10.0 (9.8;11.5)	11.0 (6.3;12.0)	0.946
Bone age – chronological age difference (years)	-1.2 (-2.2;-0.4)	-1.0 (-1.8;-0.6)	-1.7 (-2.6;-0.4)	0.659
Height (SDS)	-1.90 (-2.38;-1.26)	-2.04 (-2.38;-1.81)	-1.57 (-2.38;-0.93)	0.096
BMI (SDS)	-0.64 (-1.11;0.22)	-0.52 (-0.88;-0.43)	-0.81 (-1.51;-0.25)	0.148
GHD etiology				
- CPHD	4 (10%)	1 (5%)	3 (15%)	0.395
- dGHD	11 (28%)	7 (35%)	4 (20%)	
- SUS	25 (62%)	12 (60%)	13 (65%)	
Reason for somatrogon choice				**0.040**
- Management simplification	13 (33%)	9 (43%)	4 (20%)	
- Multiple treatments	11 (28%)	6 (29%)	6 (30%)	
- Needle phobia	9 (23%)	6 (29%)	3 (15%)	
- Poor adherence	5 (13%)	0 (0%)	5 (25%)	
- Behavioral problems	2 (5%)	0 (0%)	2 (10%)	
Somatrogon dose (mg/week)	20.3 (15.8;27.3)	22.5 (18.0;26.3)	19.8 (13.0;28.0)	0.425
Somatrogon dose (mg/kg/week)	0.66 (0.65;0.66)	0.66 (0.65;0.66)	0.66 (0.64;0.66)	0.989

Data are presented as number and percentage for categorical variables and as median (interquartile range) for continuous variables. Significant p-values are highlighted in bold.

**Figure 1:**
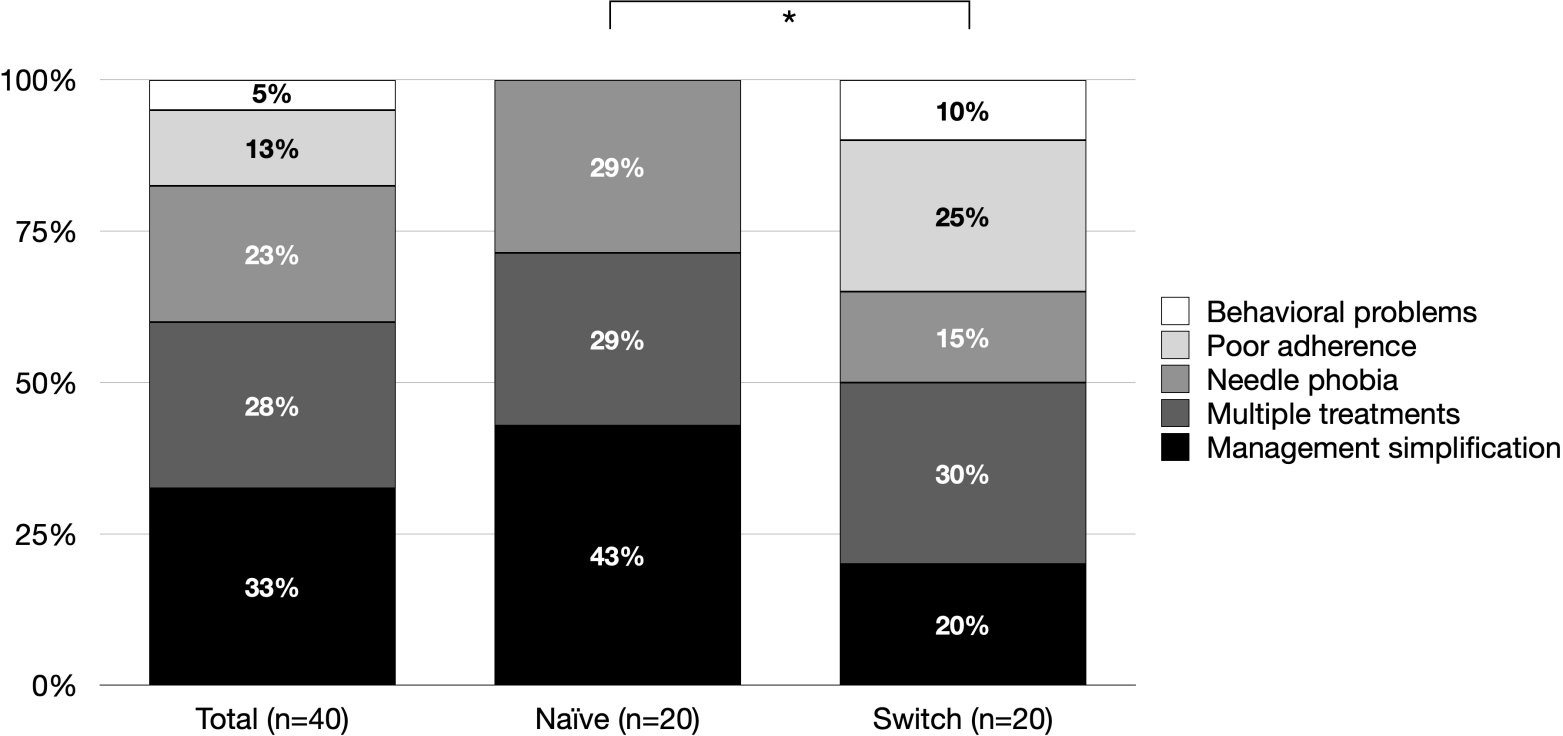
Reported reasons for choosing somatrogon treatment in the overall cohort and in the Naïve and Switch subgroups. Asterisk indicates significant difference: *p < 0.05.

The reported reasons for choosing somatrogon were family management simplification (32%), concurrent multiple treatments (28%), needle phobia (23%), poor adherence (12%), and behavioral problems (5%). These reasons differed significantly between groups (*p* = 0.040): poor adherence and behavioral problems were reported exclusively in the Switch group (25% and 10%, respectively), whereas the Naïve group more frequently cited management simplification (45%) (**Figure 1**).

No other significant differences were found between the two groups in terms of GHD etiology, sex distribution, pubertal status, chronological age, bone age, difference between bone and chronological age, height SDS, BMI SDS, or somatrogon dose at baseline. Consistently with previous rhGH exposure in the Switch group, which had a median treatment duration of 1.7 years and a median rhGH dose of 28.9 mcg/kg/day before transitioning, baseline IGF-1 levels were higher in the Switch group (-0.60 SDS [-1.15;0.92]) than in the Naïve group (-1.05 SDS [-1,73;-0.58]), although the difference did not reach statistical significance (*p* = 0.060).

Follow-up data were available for all 40 patients at 6 months (100%), for 33 at 12 months (83%), 18 at 18 months (45%), and 12 at 24 months (30%). Data on auxological and biochemical parameters for the entire cohort are presented in **Table 2**. **Table 3** report the same parameters for the Naïve group, and **Table 4** for the Switch group, including data from the last 6 months of somatropin treatment prior to the initiation of somatrogon.

**Table 2:** Longitudinal changes in auxological and biochemical parameters over 24 months of somatrogon treatment for the entire cohort.

Variables	Baseline	6 months	P-value (6 m vs. baseline)	12 months	P-value (12 m vs. 6 m)	18 months	P-value (18 m vs. 12 m)	24 months	P-value (24 m vs. 18 m)
N (%)	40 (100%)	40 (100%)		33 (83%)		18 (45%)		12 (30%)	
Chronological age (years)	11.4 (10.0;12.9)	12.1 (8.6;13.2)	**<0.001**	12.5 (11.0;13.5)	**<0.001**	13.0 (12.2;13.7)	**<0.001**	13.1 (11.9;13.5)	**<0.001**
Bone age (years)	10.5 (7.9;12.0)	11.5 (8.0;12.5)	**<0.001**	11.0 (9.5;13.0)	**0.019**	12.0 (11.4;13.0)	0.071	12.5 (11.8;14.0)	**0.015**
Bone age – chronological age difference (years)	-1.2 (-2.2;-0.4)	-1.1 (-2.4;-0.2)	0.119	-1.4 (-2.2;0.3)	0.443	-0.8 (-1.8;0.3)	0.375	-0.5 (-1.1;0.5)	1.000
Height (SDS)	-1.90 (-2.38;-1.26)	-1.65 (-2.20;-1.19)	**<0.001**	-1.50 (-2.17;-0.83)	**0.007**	-1.50 (-1.85;-0.77)	**0.002**	-1.41 (-1.83;-1.21)	0.910
Δ Height (SDS)		0.19 (0.06;0.33)	–	0.29 (0.15;0.49)	–	0.46 (0.28;0.83)	–	0.36 (0.02;0.71)	–
BMI (SDS)	-0.64 (-1.11;0.22)	-0.23 (-0.83;0.11)	**<0.001**	-0.22 (-1.00;0.21)	0.367	-0.20 (-0.81;0.41)	0.256	-0.05 (-0.66;0.66)	0.638
Δ BMI (SDS)		0.18 (-0.04;0.46)	–	0.30 (0.03;0.59)	–	0.11 (-0.08;0.38)	–	0.09 (-0.27;0.31)	–
Somatrogon dose (mg/week)	20.3 (15.8;27.3)	22.0 (17.8;26.0)	0.205	23.0 (17.0;29.0)	**<0.001**	25.0 (18.9;30.0)	**0.009**	26.0 (20.3;32.8)	**0.036**
Somatrogon dose (mg/kg/week)	0.66 (0.65;0.66)	0.63 (0.57;0.66)	**0.001**	0.63 (0.56;0.65)	0.741	0.62 (0.56;0.65)	0.899	0.63 (0.57;0.66)	0.733
Dose change from previous visit (%)		4 (0;13)	–	5 (0;14)	0.695	2 (0;8)	0.402	4 (0;13)	0.722
IGF-1 (SDS) chronological age	-1.28 (-2.09;-0.14)	0.51 (-0.17;0.93)	**0.002**	0.12 (-0.50;0.79)	0.359	0.25 (-0.26;0.70)	0.375	0.47 (-0.47;1.06)	0.496
Δ IGF-1 (SDS) chronological age		0.99 (-0.01;2.09)	–	0.24 (-0.42;1.82)	–	0.84 (-0.27;1.90)	–	-0.07 (-0.92;1.54)	–
IGF-1 (SDS) bone age	-0.73 (-1.54;0.36)	0.84 (-0.02;1.41)	**0.006**	0.76 (-0.39;1.55)	0.978	0.62 (-0.03;0.81)	0.695	0.84 (-0.29;1.08)	0.476
Δ IGF-1 (SDS) bone age		1.12 (-0.00;2.23)	–	0.25 (-0.13;2.02)	–	1.14 (-0.14;1.66)	–	0.31 (-0.67;1.23)	–

Data are presented as median (interquartile range). Δ indicates the variation in SDS from baseline. Statistically significant p-values (p < 0.05) are shown in bold. SDS, standard deviation score; BMI, body mass index; IGF-1, insulin-like growth factor 1.

**Table 3:** Longitudinal changes in auxological and biochemical parameters over 24 months of somatrogon treatment for the naïve group.

Variables	Baseline	6 months	P-value (6 m vs. baseline)	12 months	P-value (12 m vs. 6 m)	18 months	P-value (18 m vs. 12 m)	24 months	P-value (24 m vs. 18 m)
N (%)	20 (100%)	20 (100%)		17 (85%)		9 (45%)		3 (15%)	
Chronological age (years)	11.6 (10.8;13.1)	12.3 (11.0;13.6)	**<0.001**	12.2 (11.4;13.5)	**<0.001**	13.2 (12.1;13.8)	**0.004**	12.5 (11.2;13.0)	**0.004**
Bone age (years)	10.0 (9.8;11.5)	11.0 (9.3;12.0)	**0.006**	11.0 (10.3;12.5)	0.269	12.0 (11.0;12.0)	1.000	12.0 (11.3;13.0)	0.371
Bone age – chronological age difference (years)	-1.0 (-1.8;-0.6)	-0.6 (-1.7;-0.1)	0.553	-0.8 (-2.2;0.1)	0.875	-1.1 (-1.8;-0.2)	1.000	-0.4 (-0.0;0.5)	0.750
Height (SDS)	-2.04 (-2.38;-1.81)	-1.80 (-2.25;-1.48)	**<0.001**	-1.48 (-2.08;-1.09)	**0.007**	-1.13 (-1.62;-0.52)	**0.004**	-0.21 (-0.51;-0.19)	0.500
Δ Height (SDS)		0.19 (0.10;0.33)	–	0.37 (0.21;0.66)	–	0.81 (0.49;1.01)	–	1.24 (0.82;1.42)	–
BMI (SDS)	-0.52 (-0.88;-0.43)	-0.20 (-0.51;0.47)	0.062	-0.18 (-0.46;0.21)	0.356	-0.20 (-0.55;0.35)	0.074	-0.16 (-0.25;0.23)	0.250
Δ BMI (SDS)		0.20 (-0.12;0.44)	–	0.32 (-0.02;0.62)	–	0.19 (-0.06;-05;0.56)	–	0.23 (0.13;0.39)	–
Somatrogon dose (mg/week)	22.5 (18.0;26.3)	23.5 (18.8;27.8)	**0.049**	24.0 (19.0;30.0)	0.081	28.5 (18.5;30.0)	0.098	29.0 (22.0;32.0)	1.000
Somatrogon dose (mg/kg/week)	0.66 (0.65;0.66)	0.65 (0.61;0.66)	0.163	0.63 (0.56;0.65)	0.263	0.58 (0.56;0.62)	0.820	0.59 (0.52;0.59)	0.750
Dose change from previous visit (%)		7 (0;14)	–	1 (0;9)	0.187	0 (0;7)	0.529	0 (0;8)	1.000
IGF-1 (SDS) chronological age	-1.96 (-2.55;-0.74)	0.93 (-0.80;1.47)	**0.004**	0.19 (-0.36;1.65)	1.000	-0.16 (-0.36;0.74)	0.250	1.71 (1.56;1.87)	1.000
Δ IGF-1 (SDS) chronological age		2.36 (1.95;2.53)	–	2.37 (-1.82;3.32)	–	1.90 (1.71;2.27)	–	2.37 (2.36;2.37)	–
IGF-1 (SDS) bone age	-1.05 (-1.73;-0.58)	1.41 (-0.91;1.66)	**0.004**	1.15 (0.36;1.55)	0.875	0.60 (0.38;0.74)	0.250	1.24 (0.97;1.51)	1.000
Δ IGF-1 (SDS) bone age		2.23 (2.06;2.44)	–	2.55 (1.47;2.95)	–	1.39 (1.23;2.43)	–	1.91 (1.63;2.19)	–

Data are presented as median (interquartile range). Δ indicates the variation in SDS from baseline. Statistically significant p-values (p < 0.05) are shown in bold. SDS, standard deviation score; BMI, body mass index; IGF-1, insulin-like growth factor 1.

**Table 4:** Longitudinal changes in auxological and biochemical parameters for the last 6 months of somatropin treatment and over 24 months of somatrogon treatment for the switch group.

Variables	-6 months	P-value (6 m vs. baseline)	Baseline	6 months	P-value (6 m vs. baseline)	12 months	P-value (12 m vs. 6 m)	18 months	P-value (18 m vs. 12 m)	24 months	P-value (24 m vs. 18 m)
N (%)	20 (100%)		20 (100%)	20 (100%)		16 (80%)		9 (45%)		9 (45%)	
Chronological age (years)	10.9 (6.6;12.1)	**<0.001**	11.4 (7.2;12.8)	12.1 (8.1;13.2)	**<0.001**	12.5 (10.1;13.5)	**0.004**	13.0 (12.4;13.2)	**0.004**	13.2 (12.9;13.5)	**0.004**
Bone age (years)	10.5 (5.8;11.5)	**0.003**	11.0 (6.3;12.0)	11.5 (6.8;12.6)	**<0.001**	11.3 (8.4;13.0)	0.053	13.0 (11.8;13.8)	0.129	12.8 (11.9;14.0)	**0.037**
Bone age – chronological age difference (years)	-0.8 (-2.1;-0.35)	**0.046**	-1.7 (-2.6;-0.4)	-1.4 (-2.7;0.4)	0.159	-1.5 (-2.1;0.5)	0.465	-0.4 (-1.2;0.4)	0.469	-0.5 (-1.6;0.1)	0.688
Height (SDS)	-1.67 (-2.49;-1.03)	**<0.001**	-1.57 (-2.38;-0.93)	-1.29 (-1.98;-0.78)	**<0.001**	-1.61 (-2.21;-0.74)	0.443	-1.72 (-1.95;-1.39)	0.359	-1.67 (-2.00;-1.24)	0.820
Δ Height (SDS)	0.15 (0.04;0.27)	0.622		0.17 (0.05;0.30)	–	0.26 (0.10;0.30)	–	0.27 (0.00;0.43)	–	1.24 (-0.02;0.37)	–
BMI (SDS)	-0.75 (-1.73;-0.07)	0.615	-0.81 (-1.51;-0.25)	-0.63 (-1.27;-0.04)	**0.007**	-0.47 (-1.27;0.321)	0.632	-0.19 (-1.00;1.04)	0.834	0.06 (-1.06;0.79)	0.953
Δ BMI (SDS)	-0.02 (-0.08;0.34)	0.167	–	0.18 (-0.11;0.50)	–	0.30 (0.13;0.48)	–	0.07 (-0.09;0.37)	–	0.05 (-0.31;0.23)	–
Somatrogon dose (mg/week)	–	–	19.8 (13.0;28.0)	20.0 (13.8;25.0)	0.972	23.0 (14.3;27.3)	**0.004**	23.0 (20.0;32.0)	0.059	24.0 (22.0;32.0)	0.058
Somatrogon dose (mg/kg/week)	–	–	0.66 (0.64;0.66)	0.60 (0.54;0.65)	**0.002**	0.62 (0.58;0.66)	0.117	0.63 (0.62;0.65)	0.910	0.65 (0.62;0.67)	0.359
Dose change from previous visit (%)	–	–	–	0 (-2;13)	–	8 (0;15)	0.074	5 (0;13)	0.673	8 (0;13)	0.933
IGF-1 (SDS) chronological age	-0.54 (-1.33;0.60)	0.985	-0.35 (-1.79;0.82)	0.08 (-0.48;0.80)	0.348	-0.16 (-1.06;0.59)	0.278	0.59 (-0.12;0.69)	1.000	0.02 (-0.76;0.90)	0.219
Δ IGF-1 (SDS) chronological age	-0.03 (-0.26;0.27)	0.611		0.33 (-0.26;0.92)	–	-0.20 (-0.82;0.38)	–	-0.49 (-0.74;0.49)	–	-0.17 (-1.40;0.50)	–
IGF-1 (SDS) bone age	-0.58 (-0.93;1.00)	0.984	-0.60 (-1.15;0.92)	0.21 (-0.53;0.84)	0.821	0.56 (-0.85;1.05)	0.898	0.68 (-0.27;1.35)	0.469	0.84 (-0.69;0.96)	0.375
Δ IGF-1 (SDS) bone age	-0.02 (-0.24;0.19)	0.818		0.34 (-0.31;1.07)	–	-0.01 (-0.94;0.36)	–	-0.012 (-0.28;0.51)	–	0.05 (-0.96;0.56)	–

Data are presented as median (interquartile range). Δ indicates the variation in SDS from baseline. Statistically significant p-values (p < 0.05) are shown in bold. SDS, standard deviation score; BMI, body mass index; IGF-1, insulin-like growth factor 1.

Chronological age and bone age increased progressively and significantly over time, as expected, while the difference between bone age and chronological age did not change significantly over time, suggesting a relatively stable maturation pattern under treatment.

**Figure 2:**
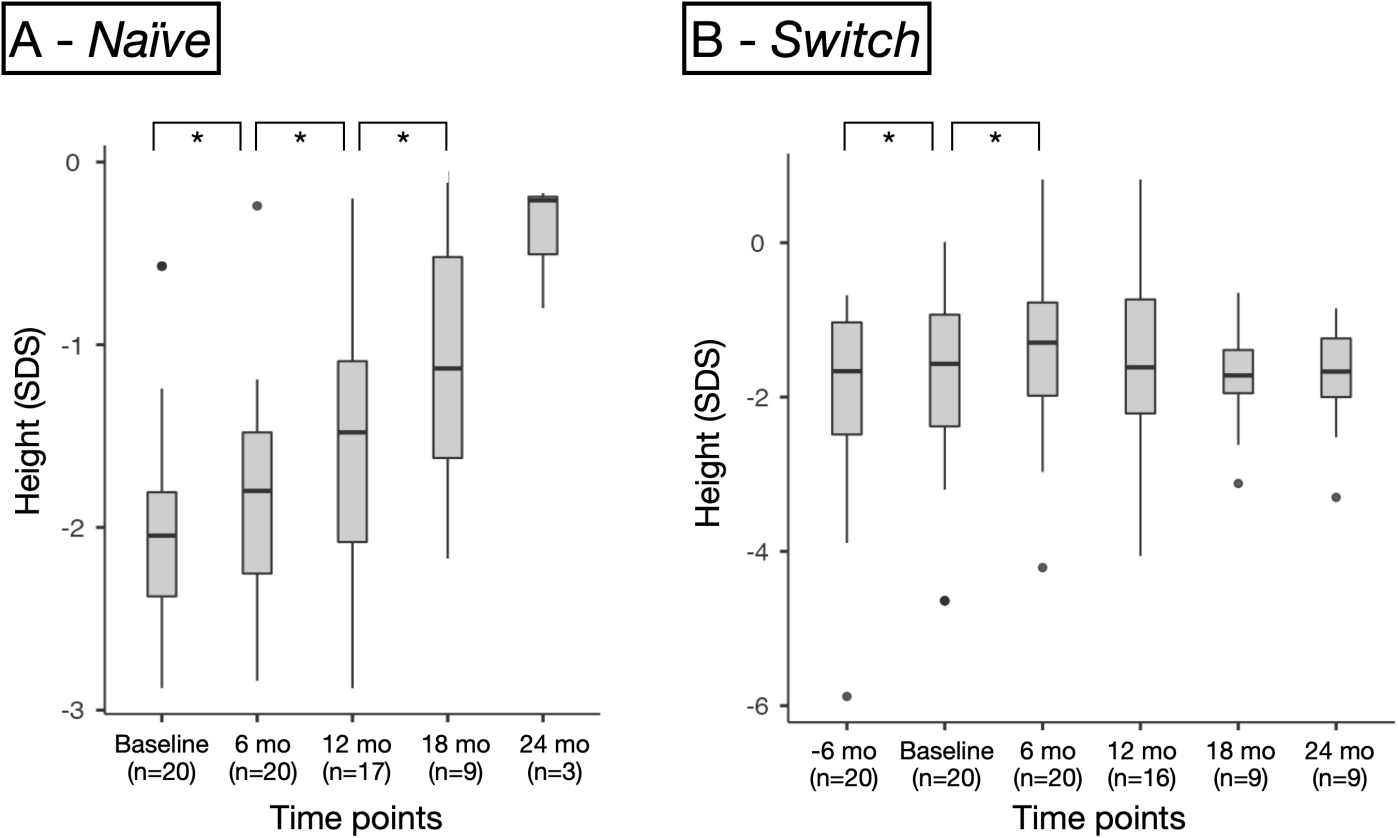
Changes in height standard deviation score (SDS) for **(A)** the naïve group, and **(B)** the switch group. Boxes represent the interquartile range (IQR), horizontal lines indicate the median, and whiskers extend to 1.5×IQR. Dots represent outliers. Asterisks indicate significant differences: *p < 0.01.

Height SDS improved significantly between baseline and 6 months, both in the Naïve group (*p* < 0.001, Δ = +0.19 [0.10;0.33]) and the Switch group (*p* < 0.001, Δ = 0.17 SDS [0.05;0.30]) *(***Figure 2***)*, with no significant difference between groups (*p* = 0.499). Further significant gains were observed at 12 months (*p* = 0.007, Δ =+0.37 [0.21;0.66]) and 18 months (*p* = 0.002, Δ =+0.81 [0.49;1.01]) only in the Naïve group (**Table 3**, **Figure 2A**), while the Switch group showed stabilization of height SDS after the initial 6-month improvement, with no further significant changes thereafter (**Table 4**, **Figure 2B***)*. By 18 months, the increase in height SDS was significantly greater in the Naïve group compared to the Switch group (*p* = 0.027), and this difference persisted at 24 months when comparing absolute height SDS values (*p* = 0.009). Although height SDS significantly improved also during the last 6 months of somatropin treatment in the Switch group (median Δ = +0.15 [0.04; 0.27]), this increase appeared lower than that observed during the first 6 months of somatrogon treatment; however, the difference was not statistically significant (p = 0.622) (**Figure 2B**).

To explore potential predictors of growth response, we performed a multivariable linear regression analysis using Δ height SDS at 6 and 12 months as dependent variables. At 6 months, the model explained 40% of the variance (R² = 0.401), with the difference between bone age and chronological age at baseline emerging as the only statistically significant predictor (p = 0.043), indicating that delayed skeletal maturation was associated with greater short-term growth. The 12-month model showed even stronger predictive power, explaining 57% of the variance (R² = 0.570). Although no variables reached conventional statistical significance, pubertal status and baseline somatrogon dose showed borderline associations with Δ height SDS (both p = 0.060), suggesting a possible role of maturational status and treatment intensity in shaping mid-term response. The difference between bone age and chronological age again showed a negative trend, consistent with the 6-month findings.

BMI SDS showed a modest but significant increase from baseline to 6 months only in the Switch group (*p* = 0.007) *(***Table 4***)*. No significant changes in BMI SDS were observed in the Naïve group at any time point (**Table 3**).

IGF-1 SDS (both for chronological and bone age) increased significantly from baseline to 6 months in the Naïve group only (*p* = 0.004, Δ = +2.36 [1.95;2.53]), while no significant change was observed in the Switch group. Between-group comparisons confirmed a significantly greater Δ IGF-1 SDS in the Naïve group compared to the Switch group at 6 months (*p* < 0.001 for chronological age; *p* = 0.001 for bone age). This difference persisted over time: at 12 and 18 months, Δ IGF-1 SDS remained significantly higher in the Naïve group (*p* < 0.001 and *p* = 0.003, respectively, for chronological age; *p* = 0.002 and *p* = 0.031 for bone age). In contrast, IGF-1 SDS values in the Switch group remained stable and within the normal range throughout follow-up *(***Table 4***)*.

Somatrogon was initiated at a dose of 0.66 mg/kg/week (± 5%) in 36 patients (90%). In 2 patients, the dose was slightly below -5% to avoid multiple weekly injections, adjusting to a submultiple of the 60 mg formulation. One patient with panhypopituitarism secondary to craniopharyngioma started at 0.45 mg/kg/week and maintained the same dose for the first year of treatment due to an excellent growth response. Another patient, who had concomitant GHD and stage III chronic renal insufficiency (CRI), began at 0.33 mg/kg/week, with the dose gradually increased to 0.51 mg/kg/week at 18 months.

**Figure 3:**
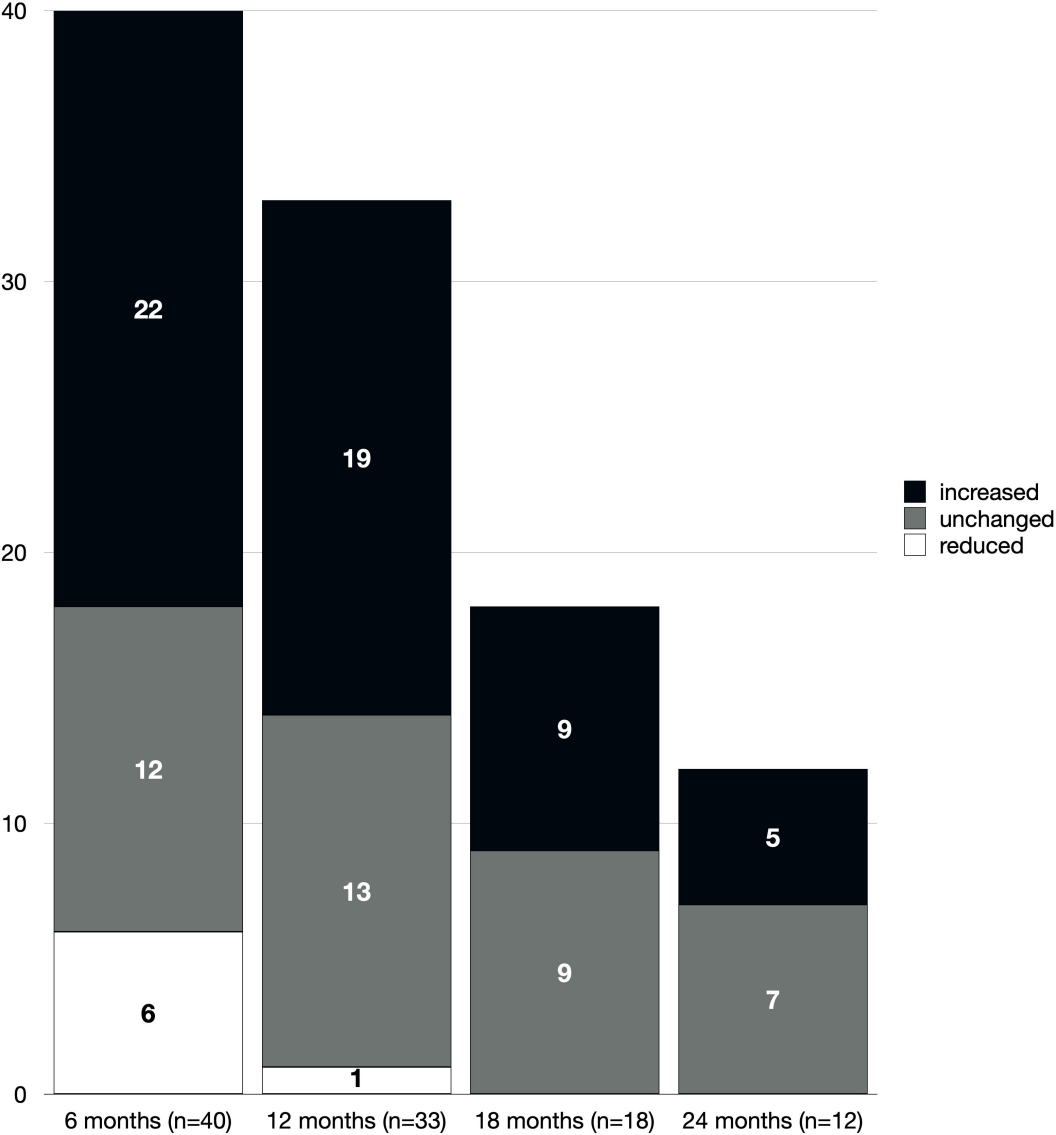
Changes in somatrogon dose over time. Stacked bars represent the number of patients at each follow-up interval whose weekly somatrogon dose was increased (black), unchanged (gray), or reduced (white) compared to the previous visit.

Regarding somatrogon dose adjustments, a reduction (median –21%) was required in 6 patients (13%) within the first 6 months of treatment due to IGF-1 levels exceeding +2 SDS. Among these, two had CPHD —one congenital (dose reduced to 0.37 mg/kg/week) and one secondary to total body irradiation for neuroblastoma (reduced to 0.54 mg/kg/week); one had dGHD (empty sella) and 2 had SUS. No further dose reductions were needed during the subsequent follow-up period, except for one naïve SUS patient in whom an IGF-1 receptor (*IGF1R*) mutation was suspected due to a clinical phenotype characterized by birth weight of –2.50 SDS, birth length of –3.19 SDS, microcephaly (–3.40 SDS), height of –2.31 SDS, and a baseline IGF-1 SDS of 0.45. During treatment, IGF-1 SDS remained between +2.5 and +3 despite progressive dose reductions, with the somatrogon dose lowered to 0.23 mg/kg/week after one year of therapy. Most patients either required a dose increase (55/103 visits, 53%) or maintained a stable dose (41/103 visits, 40%), with the frequency of adjustments progressively decreasing over time *(***Figure 3***)*. The reasons for dose increases were either suboptimal growth rate (height velocity <0 SDS, as for clinical practice) (n=32, 58%) or the need for adjustment due to weight gain (n=23, 42%).

Overall, the somatrogon dose (mg/kg/week) showed a slight decrease over time, with a significant reduction from baseline to 6 months (*p* < 0.001) in the Switch group only *(***Table 4***)*, while subsequent changes were not statistically significant.

Regarding side effects, 4 (11%) reported adverse events in the first 6 months of treatment: 3 experienced injection site pain that gradually improved, and 1 reported injection site erythema, which resolved with oral cetirizine. No cases of lipohypertrophy or lipoatrophy were observed. Additionally, 6 families in the switch group (30%) spontaneously reported qualitative improvements in quality of life, such as resolution of injection-related anxiety and fewer behavioral disturbances surrounding the administration. Notably, in one case, a child with neurodevelopmental comorbidities who had previously required restraint for daily injections began to accept the weekly dose calmly, which was described by the caregiver as ‘life-changing’. In another case, the parents observed that their child’s insomnia resolved after switching to weekly somatrogon, attributing the sleep disturbance to the previous routine of daily bedtime injections.

No patient discontinued somatrogon therapy or reverted to somatropin during the observation period.

## Discussion

To our knowledge, this is the first real-world study on the use of somatrogon, providing a comprehensive overview of its effectiveness, safety, and dose management in a diverse cohort of pediatric patients with GHD. Over a follow-up period of up to 24 months, somatrogon demonstrated sustained effectiveness in promoting linear growth, a favorable safety profile, and acceptable IGF-1 control, thereby confirming and extending the findings of previous clinical trials conducted in controlled settings (5–7). Notably, it includes for the first time patients who were switched from daily somatropin, a group not represented in phase III trials.

Although a significant increase in height SDS was observed and considered an acceptable response to treatment (15), the gains at 12 months in the 17 GH-naïve patients in our cohort (+0.37 SDS) were lower than those reported in phase III clinical trials. Specifically, the Japanese study reported a mean increase of +0.94 SDS (6), while the global trial showed a gain of +0.92 SDS at 12 months (16). This discrepancy likely reflects the clinical heterogeneity of our real-world population, which differs substantially from the strictly selected, prepubertal, treatment-naïve cohorts enrolled in randomized controlled trials.

Our cohort included patients with a broader age range, prior GH exposure, comorbidities, and even pubertal onset – features that are commonly encountered in routine clinical practice. Although the sample size did not allow for a formal subgroup analysis by pubertal status, no safety concerns emerged in pubertal patients, suggesting that somatrogon may be a feasible option even beyond the prepubertal stage. Nonetheless, older age at treatment initiation, early puberty, variable adherence, and less intensive monitoring may have contributed to the more modest growth response observed in our real-world setting (17).

Interestingly, despite expectations of reduced responsiveness (18), no significant differences in height SDS gain were observed between naïve (Δ = +0.21 SDS) and switch patients (Δ = +0.19 SDS) during the first 6 months of somatrogon therapy. This finding is consistent with previous real-world evidence using daily GH, which also reported comparable growth responses between GH-naïve and previously treated children receiving somatropin (19). In our cohort, switch patients had previously received daily rhGH for a median of 1.7 years (median dose: 28.9 mcg/kg/day), yet their early growth response to somatrogon was similar – and slightly greater than in the 6 months preceding the switch – suggesting that reduced adherence to daily injections may have limited prior treatment effectiveness.

Nonetheless, longitudinal analysis revealed divergent patterns over time. By 18 months, height SDS gains were significantly greater in the naïve group compared to the switch group, and this difference remained evident at 24 months when comparing absolute height SDS values. Likewise, IGF-1 SDS increases were consistently higher in the naïve group at 6, 12, and 18 months, suggesting a more sustained biochemical response in patients with no prior GH exposure. In contrast, the switch group showed early stabilization of both height and IGF-1 SDS, with no significant changes beyond the initial 6 months. These findings support the notion that somatrogon’s growth-promoting effects are more pronounced and durable in treatment-naïve individuals, likely due to greater intrinsic sensitivity to GH. This difference is not unexpected, since GH-naïve patients usually experience an initial catch-up growth phase after starting therapy, which is attenuated in previously treated patients. Therefore, the better auxological and biochemical response observed in our naïve cohort may largely reflect this well-known phenomenon rather than a differential efficacy of somatrogon itself.

Despite these differences, cumulative height SDS gain at 24 months reached +1.24 SDS in the overall cohort, demonstrating that somatrogon maintains substantial growth-promoting potential with continued use—even in a clinically diverse and complex real-world population.

In our regression analyses, the difference between bone age and chronological age at baseline consistently emerged as a relevant predictor of growth response, achieving statistical significance at 6 months and showing a similar trend at 12 months. This supports previous findings that delayed skeletal maturation may indicate greater growth potential during GH therapy (20).

At 12 months, the regression model captured over half of the variance in Δ height SDS, with pubertal status and baseline somatrogon dose approaching statistical significance. These findings suggest that individual clinical characteristics – such as maturity at treatment initiation and dose modulation – may contribute meaningfully to growth outcomes in real-world settings. Altogether, the models highlight the multifactorial nature of growth response and the importance of personalized GH management, particularly when using long-acting formulations.

In our cohort, the modest BMI SDS increase was limited to the Switch group during the first 6 months of somatrogon therapy, while no significant changes were observed in the Naïve group. Notably, phase III trials of somatrogon did not report changes in BMI SDS, leaving a gap in our understanding of the metabolic effects of LAGH formulations. Interestingly, while some studies on daily somatropin reported stable or even reduced BMI SDS during the first year of treatment (21), others have shown a significant increase in BMI SDS, particularly in children with GHD (9, 22). While this trend likely reflects physiological weight gain during catch-up growth, it also highlights the importance of monitoring metabolic parameters over time.

Importantly, IGF-1 SDS levels increased significantly during the first 6 months of treatment exclusively in the treatment-naïve group (+2.36 SDS), as expected, with no significant changes observed in the Switch group. Although baseline IGF-1 SDS was slightly higher in the Switch group, the Naïve group showed a significantly greater biochemical response at all time points. Following this initial rise, IGF-1 levels remained stable over time, in line with previous clinical trial data (5–7). These findings are consistent with a recent network meta-analysis (23), which showed that somatrogon had the highest increase in mean IGF-1 SDS among all long-acting GH formulations, reinforcing its capacity to elicit robust biochemical responses while remaining within the recommended safety margins. In our cohort, IGF-1 exceeded +2 SDS in 13% of patients during the early phase of treatment, prompting temporary dose reductions. Notably, the proportion of patients requiring dose reductions was comparable to that reported in previous reports (11% in the international study overall, and 18.2% in the Japanese trial at 6 months) (5, 6), and no discontinuations occurred.

Regarding dose evolution, our findings illustrate a typical pattern: a modest decrease in mg/kg/week dosage over time (likely due to weight gain), with a corresponding increase in absolute weekly dose in milligrams. This reflects expected growth-related changes in body mass rather than treatment inefficacy. Most patients required either no adjustment or an increase in dose due to weight gain or suboptimal growth response. These real-world dosing trends align with clinical trial protocols, which recommend dose adjustments based on weight and IGF-1 levels​.

Our report also contributes novel data on skeletal maturation, showing a stable bone age–chronological age difference over time. This finding aligns with the results of the recent network meta-analysis, which found comparable effects of long-acting and daily GH formulations on the bone age to chronological age ratio (23), supporting the skeletal safety of LAGH in routine clinical practice.

While the number of cases is too small (n = 3) to draw definitive conclusions, we hypothesize that in patients with panhypopituitarism or severe GHD, a lower somatrogon dose than the standard 0.66 mg/kg/week may be sufficient. In fact, the first two patients with panhypopituitarism treated with somatrogon – one of whom was a cancer survivor – required early dose reductions due to IGF-1 levels exceeding +2 SDS, with final doses adjusted to 0.37 and 0.54 mg/kg/week, respectively. A third patient with GHD secondary to craniopharyngioma, who started treatment at a reduced dose of 0.45 mg/kg/week, showed good linear growth and maintained IGF-1 levels within the normal range during the first year of therapy. Even though somatrogon is not currently approved for short stature associated with CRI – like other conditions such as Turner syndrome, SHOX deficiency, and additional indications for somatropin – one patient in our cohort with concomitant GHD was treated with somatrogon because of multitherapy. Treatment was initiated at a dose of 0.33 mg/kg/week and gradually increased to 0.51 mg/kg/week over 18 months, with no adverse effects reported. In line with consensus guidance (4), our data suggest that lower starting doses may be appropriate in specific subpopulations – such as patients with panhypopituitarism, chromosomal abnormalities, or chronic renal insufficiency – to minimize the risk of excessive IGF-1 levels. The only patient with persistently elevated IGF-1 levels (> +2 SDS) despite somatrogon dose reduction is under evaluation for a suspected *IGF1R* mutation, to be confirmed (24). In this condition, IGF-1 concentrations during GH treatment – whether daily or weekly – are typically elevated but are not associated with increased growth response or adverse events and are therefore not considered clinically concerning.

The clinical profiles observed in our cohort provide, for the first time in a real-world setting, evidence of the patient categories that we previously identified as likely to benefit from LAGH therapy (3), in line with other authors’ suggestions (4). Overall, the main categories included children and adolescents for whom treatment simplification was needed (33%) – such as those living in multiple households or frequently traveling – and those taking multiple concomitant medications (28%). In 22% of cases, somatrogon was selected to address injection-related fear and anxiety. Notably, poor adherence (25%) and behavioral issues interfering with daily injections (10%) were reported exclusively in the switch group, further supporting the rationale for transitioning these patients to LAGH.

One of the key advantages of somatrogon is its potential to improve adherence by reducing injection frequency. Although no formal adherence measures were collected in our cohort, none of the patients discontinued somatrogon or reverted to daily therapy during the observation period. In addition, approximately 30% of families in the switch group spontaneously reported improved adherence and reduced emotional distress related to injections. These observations are consistent with findings from international surveys and discrete choice experiments, which consistently indicate a strong patient and caregiver preference for weekly over daily GH therapy (25, 26). While these qualitative impressions suggest potential adherence benefits of LAGH, future studies incorporating validated adherence metrics are needed to confirm this effect.

In terms of safety, somatrogon was well tolerated, with mild injection site reactions reported in 11% of patients in the first 6 months of treatment – primarily injection site pain and erythema. While this prevalence is lower than that observed in the global (39.4% pain; 8.3% erythema) (5) and Japanese (72.7% pain) (7) phase III trials, the nature of the adverse events is consistent. This difference may reflect the real-world setting of the present study, where adverse events are not collected through systematic diaries or investigator-led queries, potentially leading to underreporting of mild, self-limited symptoms.

Overall, this real-world study provides compelling evidence that somatrogon is an effective, safe, and well-tolerated treatment option for children with GHD, including those with prior GH exposure, pubertal onset, and complex medical needs. It reinforces the consensus that long-acting GH therapy can reduce treatment burden and improve adherence, while highlighting the need for long-term prospective studies to assess metabolic outcomes and final height attainment.

Some limitations of this study include its retrospective design, the limited sample size at later time points (particularly at 24 months), and potential selection bias related to treatment choice. Additionally, quality of life improvements were reported spontaneously rather than measured using validated instruments, we did not systematically assess treatment adherence using validated tools, and final height outcomes could not yet be evaluated, suggesting areas for further prospective research. Moreover, due to the retrospective design, we were unable to collect systematic data on patients who were offered LAGH but declined the treatment option, including their reasons for refusal. This represents a potential source of selection bias and limits our understanding of real-world acceptance of LAGH in both naïve and previously treated patients. Larger cohorts with longer follow-up durations are needed to confirm long-term effectiveness and safety, as GloBE-Reg (27). Future studies should incorporate patient-reported outcome measures to better capture the patient experience.

## Conclusion

In conclusion, our real-world data confirm that once-weekly somatrogon is a safe and effective alternative to daily GH therapy in pediatric patients with GHD. While the observed growth response—particularly in GH-naïve patients—was lower than that reported in registration trials, it remained clinically meaningful, especially over extended follow-up. Somatrogon supported consistent IGF-1 control, was well tolerated, and was associated with high treatment retention. Its simplified weekly regimen may enhance treatment adherence and caregiver satisfaction, particularly in patients with complex care needs. These findings underscore the value of somatrogon in real-world settings and highlight the need for prospective studies with longer follow-up to assess long-term growth outcomes, metabolic effects, and patient-reported experiences.

## Data Availability

The raw data supporting the conclusions of this article will be made available by the authors, without undue reservation.
